# Comparing the Efficacy of Local Corticosteroid Injection, Platelet-Rich Plasma, and Extracorporeal Shockwave Therapy in the Treatment of Pes Anserine Bursitis: A Prospective, Randomized, Comparative Study

**DOI:** 10.1155/2023/5545520

**Published:** 2023-09-30

**Authors:** Wesam Gouda, Awad S. Abbas, Tarek M. Abdel-Aziz, Mohamed Z. Shoaeir, Walid Ahmed, Abdelhfeez Moshrif, Ahmed Mosallam, Mohamed Kamal

**Affiliations:** ^1^Rheumatology and Rehabilitation Department, Faculty of Medicine, Al Azhar University, Assiut, Egypt; ^2^Medicine Department, London North West University Healthcare NHS Trust, Harrow, UK; ^3^Rheumatology and Rehabilitation Department, Faculty of Medicine, Al Azhar University, Cairo, Egypt

## Abstract

**Background:**

Pes anserine bursitis (PAB) is one of the most common causes of painful knee syndromes. This study aimed at examining the efficacy of local corticosteroid injection, platelet-rich plasma (PRP) injection, and extracorporeal shock wave therapy (ESWT) as different modalities to alleviate pain and enhance function in patients with pes anserine bursitis (PAB).

**Methods:**

A prospective, randomized, comparative study was conducted on 180 patients diagnosed with chronic PAB. They were equally divided into three groups as follows: Group I received a local corticosteroid injection of 40 mg of methylprednisolone acetate/1 ml; Group II received a PRP injection; and in Group III, ESWT was used. Outcome measures included the visual analog scale (VAS), Western Ontario and McMaster Universities (WOMAC) pain score, WOMAC physical function score, and Ritchie articular index (RAI) for tenderness, which were recorded at the baseline, after 1 week, and after 8 weeks.

**Results:**

Before the application of procedures, there was a statistically significant increase in the WOMAC pain score in the local corticosteroid group compared to the PRP group and the ESWT group (*P* < 0.001). After the application of procedures, there was a statistically significant improvement in the 1-week and 8-week WOMAC pain score, WOMAC physical function score, and VAS in the local corticosteroid group in comparison to the PRP group and the ESWT group. (*P* < 0.001). Moreover, RAI for tenderness shows statistically significant improvement at 8 weeks in the local corticosteroid groups compared to the PRP groups (*P* < 0.001) and ESWT groups (*P* < 0.001). Similarly, a statistically significant difference was found between the PRP and ESWT groups (*P*=0.023).

**Conclusion:**

Our data suggest that in patients with PAB, local corticosteroid injection is more efficient than PRP injection and ESWT for reducing pain and enhancing function.

## 1. Introduction

Knee pain is one of the most prevalent reasons for seeking medical advice [1]. Pes anserine is a Latin name that means “the foot of a goose.” It is a region of the knee joint composed of the tendons of the sartorius, gracilis, and semitendinosus muscles located around 5 cm distal to the medial portion of the knee [2, 3]. Pes anserine bursitis (PAB) is one of the most common causes of painful knee syndromes. It limits physical activity and reduces a patient's quality of life. Obesity, diabetes mellitus, valgus deformity, trauma, and osteoarthritis (OA) of the knee are predisposing factors for PAB [4]. The pain of PAB is multiplied and augmented during leg crossing, standing up from a chair, and going upstairs. Patients who exhibit tenderness associated with pain over the pes anserine bursa may have pes anserine bursitis. A patient's history and clinical examination may be enough for diagnosis [5, 6]. Musculoskeletal ultrasonography can help diagnose PAB [7]. However, in most cases of pes anserine bursitis, which is diagnosed on a clinical basis, ultrasonographic features are deficient [6]. The treatment for PAB includes nonsteroidal anti-inflammatory drugs (NSAIDs), physiotherapy (PT), injections of local anesthetics, and/or corticosteroids [4]. Rehabilitation of patients with PAB includes flexibility, stretching, and strengthening exercises of the pes anserine muscles [8]. Physical modalities such as ice, phonophoresis, and iontophoresis may be effective in treating patients with PAB [9]. Platelet-rich plasma (PRP) is a plasma component generated from the patient's blood that contains a higher percentage of platelets than regular plasma. PRP can be prepared manually or with one of the several commercial PRP preparation kits [10, 11]. The high concentrations of growth factors in PRP account for its positive impact. By delivering PRP locally to the lesion, PRP injection promotes tendon and cartilage tissue regeneration, which accelerates the healing process [12, 13].

Extracorporeal shock wave therapy (ESWT) is a noninvasive therapeutic method for a variety of tendon problems, including epicondylitis, plantar fasciitis, and rotator cuff tendinitis, with a success rate estimated to be 60–80% [14]. In this randomized clinical study, we aimed at comparing the efficacy of local corticosteroid injection, PRP injection, and ESWT as different modalities to alleviate pain and enhance the function in patients with pes anserine bursitis.

## 2. Materials and Methods

### 2.1. Study Design and Participants

**Figure 1:**
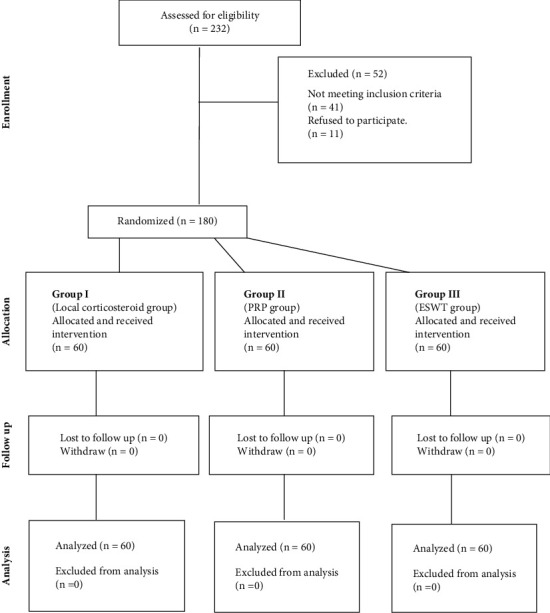
Study profile. Patients were grouped according to the therapeutic procedures received. *n*, number.

This prospective, randomized, comparative trial was performed between July 2021 and June 2022 in the Rheumatology and Rehabilitation Department at Al-Azhar University Hospital in Egypt. After assessing 232 patients for eligibility, 180 patients with chronic PAB were randomized and enrolled in the study as shown in Figure 1.

### 2.2. Ethical Consideration

The study was carried out in accordance with the ethical guidelines of the competent committee on human testing as well as the Helsinki Declaration of 1975, as amended in 1983. The Ethics Committee's approval was received for this study from the Ethics Committee and IRB of Al Azhar University School of Medicine (No. 6223/2021), Assiut City, Egypt. The study was retrospectively registered in the Pan African Clinical Trial Registry with an identifier number of PACTR202302648824229., which is a primary registry in the World Health Organization registry network. All participants were informed about the study procedures, and an informed consent form was taken from all participants for the study and data use.

### 2.3. Inclusion Criteria

All enrolled patients were clinically diagnosed with chronic PAB by a rheumatologist. The clinical diagnosis of chronic PAB was based on Larsson and Baum's criteria [15]. These include pain in the anteromedial area of the knee, especially when moving uphill or downstairs, morning discomfort and stiffness lasting more than an hour, nocturnal pain, and difficulty rising from a chair or getting out of a car. These are all symptoms of local sensitivity and edema in the pes anserine bursa. The patients who were selected were resistant to traditional treatments such as NSAIDs and PT.

### 2.4. Exclusion Criteria

Patients with blood disorders, pacemakers, paresis of the lower limbs, active malignancy or infection, pregnancy, fractures, having undergone knee surgery, or having other nonrheumatic sources of pain were among those excluded from the trial.

Patients who declined to participate in the trial, those allergic to corticosteroid injections, and those suffering from fibromyalgia were also excluded from the study.

### 2.5. Demographics and Clinical Characteristics

Age, gender, duration of symptoms, affected side, body mass index (BMI), systemic disorders, drug usage, smoking, alcohol use, trauma history, and surgical history were all recorded. (Weight (kg)/[height (m)]^2^) was used to compute BMI [16].

### 2.6. Study Procedures

The enrolled patients were divided into three groups, each including sixty patients. The groups of patients were treated by local corticosteroid injection, high PRP injection, and, lastly, ESWT.

#### 2.6.1. Group I (Local Corticosteroid Group)

Sixty patients were singly injected with local corticosteroids in the form of 40 mg of methylprednisolone acetate/1 ml (Depo-Medrol by Upjohn, USA). Under aseptic circumstances, all injection insertions were carried out by the same physician at the most tender area of the PAB with a 5 mL disposable syringe.

#### 2.6.2. Group II (PRP Group)

Sixty patients were injected with PRP. A manual method was used to prepare the PRP. ACD-A solution (2 mL) was mixed with 8 mL of venous blood and placed in sterile 10 mL tubes. In the first stage, two 10 mL tubes were centrifuged at 2000 rpm for 20 minutes. The blood was split into three layers after centrifugation as follows: the plasma layer, the second layer (containing leukocytes and platelets), and the bottom layer (consisting of erythrocytes). In the second phase, the plasma and platelet layers were transferred to two empty 10 mL tubes and centrifuged at 1500 rpm for 15 minutes. Following the last centrifugation, 2 mL of PRP was extracted with a syringe and injected into the most tender area of the PAB by the same physician.

#### 2.6.3. Group III (ESWT Group)

Sixty patients were applied for ESWT for four sessions (one session every week and each one is about 7 minutes) by using a focused probe (4 Hz frequency, 1,500 to 2,000 pulses per session), which was performed with the contact of the focused probe with the skin over PAB (6 cm below the joint line on the medial side of the knee after applying a gel).

The follow-up was carried out one week and eight weeks after the procedures were conducted to record and treat any adverse reactions and assess the clinical outcomes.

### 2.7. Postprocedure Care

Following the procedure, instructions were provided to rest the knees for 24 hours. If discomfort persisted after injection or bruising appeared, using a cold compress on the area of the injected anserine bursa was advised during the first 48 hours. Only acetaminophen is occasionally advised as a pain reliever (a dose of up to 4 g daily was approved) and should be stopped 48 hours before the subsequent evaluation visit.

### 2.8. Clinical Outcomes

All patients were reviewed by a single qualified investigator who was unaware of the patients' clinical state or group allocation, using a structured questionnaire to obtain their responses. Furthermore, every questionnaire was translated into Arabic to minimize patient misconceptions.

#### 2.8.1. Visual Analog Scale (VAS)

We employed a paper-based VAS to assess the baseline severity and changes in pain intensity caused by the therapies after 1 week and 8 weeks. Subjects self-report the level of their discomfort by placing a mark along a 10-cm line. The scale's 0 cm end denotes “no discomfort,” while the 10 cm end reflects “worst pain experienced” [17].

#### 2.8.2. Western Ontario and McMaster Universities' Osteoarthritis Index (WOMAC) for Pain and Physical Function

After 1 week and 8 weeks, each item is graded on a 4-point scale. (0: none; 1: mild; 2: medium; 3: severe; and 4: extremely severe). The survey assigns a score between 0 and 100, with higher scores indicating worse health [18].

#### 2.8.3. The Ritchie Articular Index (RAI) for Tenderness

At the baseline, after 1 week, and after 8 weeks was applied after using different modalities of treatment in this study. Tenderness was assessed using a four-point scale: 0 = no tenderness; I = patient-reported pain; 2 = patient-reported discomfort and grimaced; and 3 = patient-reported pain, winced, and withdrew the joint. The articular index in each subject was the sum of the scores from the 48 units assessed [19].

### 2.9. Statistical Analyses

The sample size was calculated a priori based on previous research [1, 3, 4, 9]. A two-sided independent *t* test was used to determine it with a significance level (*α*) of 5% and power (1 − *β*) of 80%. Accordingly, the minimum required sample size was 46 per group. Considering a possible dropout rate of 30%, the study was intended to include 180 patients. The data were analyzed using statistical software for social science (SPSS) version 24. The mean standard deviation (SD) was utilized to convey quantitative data. The frequency and proportion of qualitative data were used. The mean (average) of a discrete collection of numbers is the total of values divided by the number of values. A set's SD is a measure of its dispersion. A low SD indicates that the values are close to the set's mean, whereas a high SD indicates that the values are spread out over a wider range. The Kruskal–Wallis test (KW), chi-square test, and probability (*P* value) tests were conducted for the analyzed data. A *P* value <0.05 was considered significant. The post hoc analysis that was conducted consisted of quantitative analytical measures for multiple clinical outcome comparisons between the studied groups. The least significant difference (LSD) test was calculated for comparisons of the three treatment groups.

## 3. Results

180 patients included in this study were divided into three groups of 60 patients each, without any significant difference between these groups in regard with age (*P*=0.759), sex (*P*=0.832), BMI (*P*=0.974), or duration of symptoms (*P*=0.389) (Table 1). Before the application of procedures, there was a statistically significant (*P* < 0.001) increased WOMAC pain score in the local corticosteroid group (13.7 ± 1.8, *P* < 0.001) when compared to the PRP group (12.8 ± 2.4) and ESWT group (11.9 ± 1.5), and a statistically significant (*P*=0.004) decreased the WOMAC physical function score in the PRP group (28.9 ± 35.9) compared to the local corticosteroid group (35.9 ± 6.3). Throughout the research, no patient withdrew. There has been no patient loss due to follow-up.

**Figure 2:**
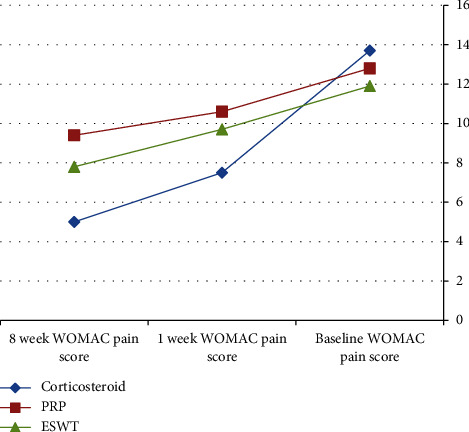
WOMAC pain score at the baseline, 1 week, and 8 weeks after the application of procedures.

**Figure 3:**
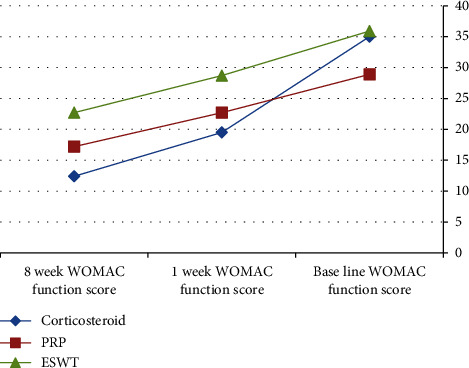
WOMAC physical function score at the baseline, 1 week, and 8 weeks after the application of procedures.

**Figure 4:**
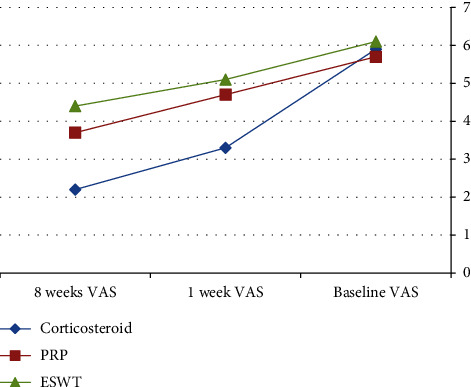
VAS at the baseline, 1 week, and 8 weeks after the application of procedures.

After the application of procedures, there was a statistically significant (*P* < 0.001) decrease in the 1-week WOMAC pain score (7.5 ± 1.9) in the local corticosteroid group in comparison to the PRP group (10.6 ± 2.5) and the ESWT group (9.7 ± 2.6). In addition, there was a statistically significant (*P* < 0.001) improvement in the 1-week WOMAC physical function score (19.5 ± 8.7) in the local corticosteroid group when compared to the PRP group (22.7 ± 6.1) and the ESWT group (28.7 ± 8.4) (Tables 2 and 3), Figure 2. Moreover, there was a statistically significant (*P* < 0.001) decreased 8-week WOMAC pain score in the local corticosteroid group (5 ± 2.7) when compared to the PRP group (9.4 ± 2.4) and ESWT group (7.8 ± 3.7) and a statistically significant (*P* < 0.001) improvement in the 8-week WOMAC physical function score in the local corticosteroid group (12.4 ± 9.4) when compared to the PRP group (17.2 ± 5.4) and the ESWT group (7.8 ± 3.7) (Tables 4 and 5), Figure 3. In regard with the VAS, there were no significant differences (*P*=0.636) among the studied groups in the baseline VAS. After the application of procedures, the intergroup comparisons showed that there was a statistically significant (*P* < 0.001) decrease in 1-week VAS in the local corticosteroid group (3.3 ± 0.8) when compared with the PRP group (4.7 ± 1.4) and the ESWT group (5.1 ± 1.5). Also, there was a statistically significant (*P* < 0.001) decrease in 8-week VAS in the local corticosteroid group (2.2 ± 0.5) compared with the PRP group (3.7 ± 1.1) and the ESWT group (4.4 ± 1.3) (Tables 6 and 7), Figure 4. When it comes to RAI for tenderness, it shows statistically significant improvement at 8 weeks in the local corticosteroid groups compared to the PRP groups (*P* < 0.001) and the ESWT groups (*P* < 0.001). Similarly, a statistically significant difference (*P*=0.023) was found between the PRP and ESWT groups (Table 8).

## 4. Discussion

PAB is one of the most common causes in the differential diagnosis of inferomedial knee pain [3]. It leads to functional disabilities and an impaired quality of life for patients. PAB is usually due to overuse of the knee joint, which leads to inflammation of the PAB [4]. Because studies on treating PAB with ESWT and PRP are very limited, this study aims at evaluating the efficacy of ESWT and PRP in the treatment of PAB versus local corticosteroid injection.

In this study, there was no significant difference in age (*P*=0.759), sex (*P*=0.832), or disease duration (*P*=0.389), in agreement with the 2 previous studies that found no statistically significant variations in the fundamental features of the PAB patients [20, 21]. When comparing the postinjection evaluation to the preinjection assessment, in all outcome measures, we identified a statistically significant improvement across the three treatment groups in this study. This was related to a considerable improvement in the RAI for tenderness in all groups. This result agreed with the study by Saba [21], who compared the efficacy of neural prolotherapy to local corticosteroid injection for the treatment of PAB and found that both groups improved significantly before and after treatment.

In the current research, the patients were assessed three times using outcome measures: WOMAC (pain and function subscale), VAS, and clinical improvement by RAI for measuring tenderness. It was determined whether there was tenderness. Patients were examined before (preinjection evaluation) and after the injection at 1 and 8 weeks (postinjection assessment). At the 8-week postinjection assessment visit, the patient had total improvement, no symptoms, and a complete return of function without tenderness on palpation. Improvement was present in 63.3% (38 patients) of the injected lower limbs with PAB in group I (local corticosteroid group), followed by group II (local PRP therapy group) and group III (ESWT therapy group) in 20% of the patients. This indicated that PRP injection was as effective as ESWT. Both treatments appear to be successful and viable solutions for treating pes anserine bursitis.

In the current study, a one-week postinjection assessment revealed that local corticosteroid injection was more effective than PRP because it reduced both the VAS and WOMAC scores of the treated subjects (decreased WOMAC pain score in the local corticosteroid group (7.5 ± 1.9) when compared to the PRP group (10.6 ± 2.5) and ESWT group (9.7 ± 2.6) and decreased VAS in the local corticosteroid group (3.3 ± 0.8) when compared to PRP). Eight weeks' postinjection assessment showed that local corticosteroid injection was the most effective treatment modality because it reduced both the VAS and WOMAC scores of the treated subjects relative to ESWT, which was more effective in improving the WOMAC pain score than PRP at 8 weeks postinjection assessment. This is consistent with the findings of a meta-analysis by Liao et al. [22], who discovered that a one-month intervention period had a significant effect on all outcomes in favor of ESWT, whereas a shorter intervention period (1 month) did not, regardless of the type of ESWT. Sarifakioglu et al. [23] investigated the efficacy of PT and local corticosteroid injection in individuals with simultaneous knee OA and pes anserine bursitis. At week 8 of therapy, the therapeutic effect was evaluated using VAS, WOMAC, and timed-up-and-go (TUG). They discovered that local corticosteroid injection and PT had comparable effectiveness. In a prospective interventional study by Yoon et al. [7], 17 patients clinically diagnosed with PAB received local corticosteroid injections, and after 2 weeks, they evaluated their efficacy based on VAS and WOMAC scores. The corticosteroid injection significantly decreased the VAS and WOMAC scores. While a study by Yasar et al. [24] concluded that corticosteroids and lidocaine are available choices to relieve pain, their results show that corticosteroids are more effective. As a result, local corticosteroid treatment may be a preferable alternative for individuals with PAB and knee OA. In our study, in the eighth week after treatment, all patients in the PRP group were examined using an RAI for tenderness, with full healing occurring in 20% (12) of patients, considerable relief in 3.3% (2%) of patients, and mild relief in 66.6% (40) of patients. To disagree with the study by Karabaş et al. [25], they found that both single-dose and double-dose local PRP are safe and efficacious therapeutic choices for PAB patients. Furthermore, in the 12th week after treatment, when all patients were evaluated using the Likert scale, full relief was shown in 22 (36.7%) patients, considerable alleviation in 25 (41.7%) patients, light relief in 4 (6.7%) patients, and increased pain was noted in 4 (6.7%) patients, while in a study by Rowicki et al. [26], a single PRP injection was given to 33 individuals with pes anserine bursitis. When compared to the baseline, there was a statistically significant improvement in VAS ratings after therapy. The Likert scale revealed that 28 patients (84.8%) healed totally or nearly completely. Moreover, one recent study has proven the efficacy of ESWT [22], studying the effect of ESWT on chronic pain reduction in patients with pes anserine bursitis. The findings indicated that ESWT might be useful in relieving pain and curing pes anserine bursitis. Based on a meta-analysis with acceptable methodological quality, Liao et al. [22] provided moderate evidence that ESWT significantly increases the treatment success rate, lowers pain, and improves functional recovery in patients with the knee's soft tissue disorders.

The current study demonstrated the first clinical study that assessed the efficacy of local corticosteroid injection in patients with chronic PAB in comparison to ESWT and PRP injection. It was hypothesized that it works by reducing vascular permeability and capillary vasodilation, which reduces inflammation. This results in a decrease in the chemotaxis of macrophages and polymorphonuclear cells as well as a decrease in the production of vasoactive kinins. Their anti-inflammatory impact is carried out via activity on a nuclear receptor targeting the glucocorticoid-responsive element, a DNA sequence, and aiding in inhibiting the production of several cytokines [27, 28]. This study has several limitations. First, the use of subjective scales such as VAS and WOMAC, which are not objective assessments, is influenced by individual views and the environment [3]. Second, there was no application of an ultrasound-guided technique while performing the injections. According to studies, guided injections have higher success rates compared to unguided injections [29]. Finally, the outcomes were analyzed in a short-term follow-up; further randomized controlled studies with longer follow-up periods are recommended.

## 5. Conclusions

Examining PAB is crucial and should not be overlooked in the knee of OA patients who complain of discomfort. Though there are some options to relieve pain, such as rest, cryotherapy, PT, and systemic NSAIDs, our results conclude that local corticosteroid injection is more effective as a treatment measure in relieving pain and improving function than PRP injection and ESWT, and therefore, it can be considered a better option in treating such cases.

## Figures and Tables

**Table 1:** Characteristics of the patients in the studied groups.

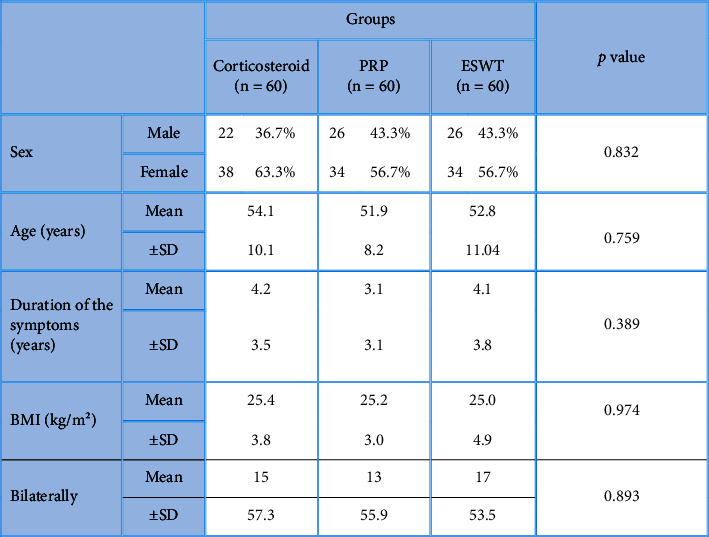

*P* value >0.05 is considered nonsignificant.

**Table 2:** Comparisons between studied groups in regard with 1-week WOMAC score (pain and physical function) after the application of procedures.

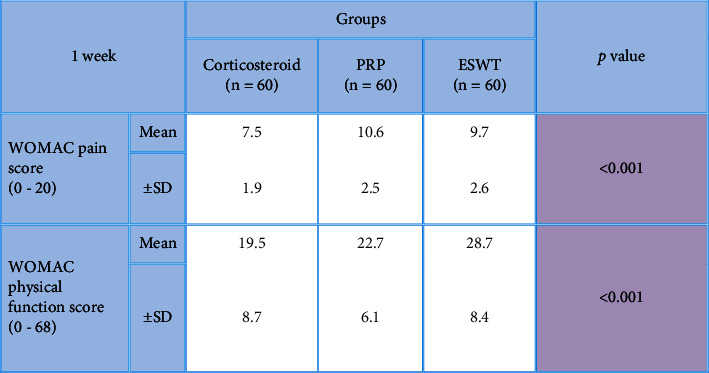

*P* value <0.001 is considered significant.

**Table 3:** Post hoc analysis for multiple comparisons between studied groups in regard with 1-week WOMAC score (pain and physical function) after the application of procedures.

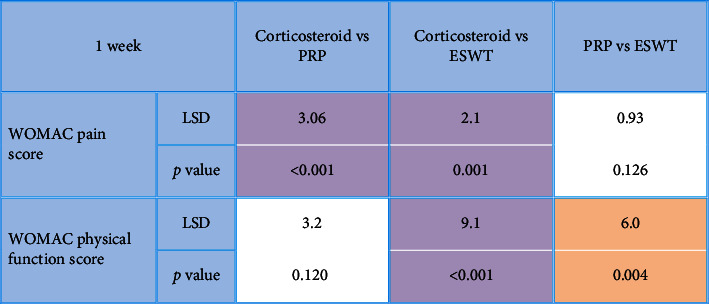

*P* value <0.05 is considered significant. *P* value >0.05 is considered nonsignificant. LSD: the least significant difference.

**Table 4:** Comparisons between studied groups in regard with 8 weeks WOMAC score (pain and physical function) after the application of procedures.

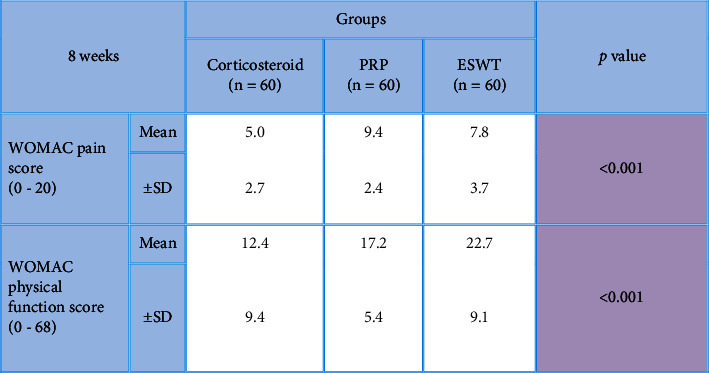

*P* value <0.001 is considered significant.

**Table 5:** Post hoc analysis for multiple comparisons between studied groups in regard with 8-week WOMAC score (pain and function) after the application of procedures.

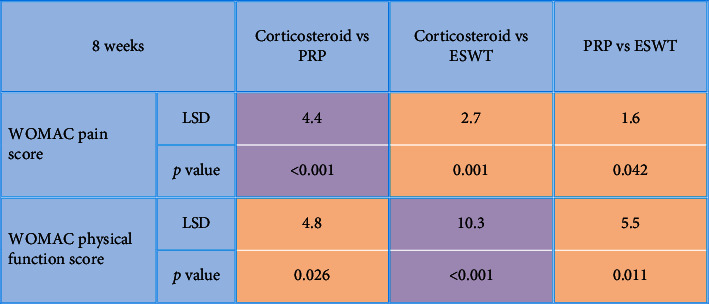

*P* value <0.05 is considered significant. LSD: the least significant difference.

**Table 6:** Comparisons between studied groups in regard with the baseline, 1-week, and 8 weeks VAS after the application of procedures.

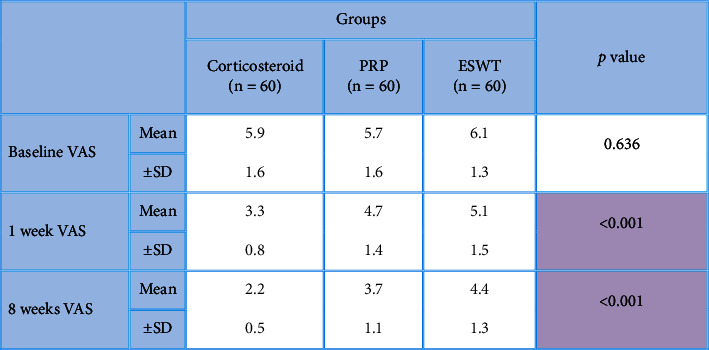

*P* value >0.05 is considered nonsignificant. *P* value <0.001 is considered significant.

**Table 7:** Post hoc analysis for multiple comparisons between studied groups in regard with the VAS score.

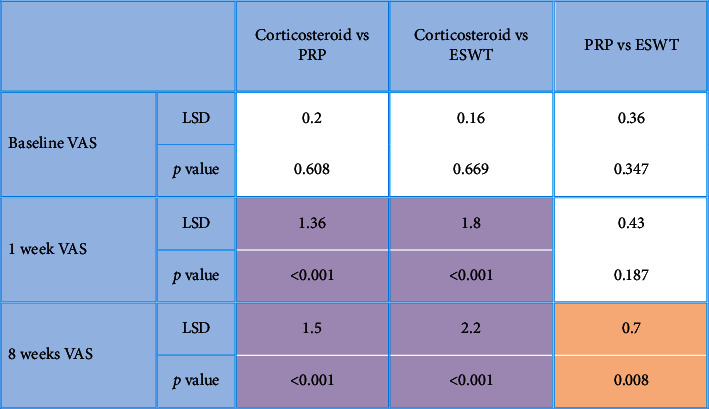

*P* value <0.05 is considered significant. *P* value >0.05 is considered nonsignificant. LSD: the least significant difference.

**Table 8:** Comparisons between studied groups in regard with the Ritchie articular index (RAI) for tenderness 8 weeks after the application of procedures.

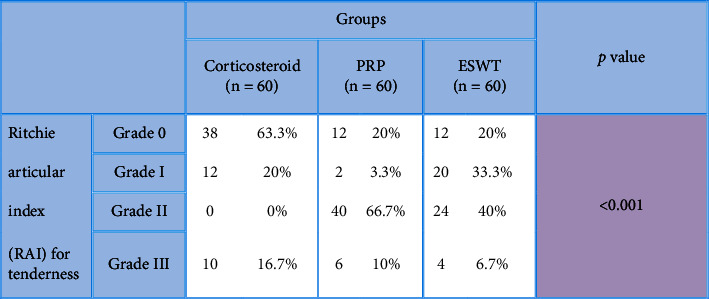

*P* value <0.001 is considered significant.

## Data Availability

The data used to support the findings of this study are available from the corresponding author upon reasonable request.

## References

[1] Lee J. H., Kim K. J., Jeong Y. G., Lee N. S., Han S. Y., Lee C. G. (2014). Pes anserinus and anserine bursa: anatomical study. *Anatomy and Cell Biology*.

[2] Alvarez-Nemegyei J. (2007). Risk factors for pes anserinus tendinitis/bursitis syndrome: a case control study. *Journal of Clinical Rheumatology*.

[3] Vega-Morales D., Esquivel-Valerio J. A., Negrete-López R., Galarza-Delgado D. Á., Garza-Elizondo M. A. (2012). Safety and efficacy of methylprednisolone infiltration in anserine syndrome treatment. *Reumatología Clínica*.

[4] Helfenstein Jr M., Kuromoto J. (2010). A síndrome anserina. *Revista Brasileira de Reumatologia*.

[5] Alvarez-Nemegyei J., Pelaez-Ballestas I., Rodriguez-Amado J., Sanin L. H., Garcia-Garcia C., Garza-Elizondo M. A. (2011). Prevalence of rheumatic regional pain syndromes in adults from Mexico: a community survey using COPCORD for screening and syndrome-specific diagnostic criteria. *Journal of Rheumatology*.

[6] Uson J., Aguado P., Bernad M., Mayordomo L., Naredo E., Balsa A. (2000). Pes anserinus tendino-bursitis: what are we talking about?. *Scandinavian Journal of Rheumatology*.

[7] Yoon H. S., Kim S. E., Suh Y. R., Seo Y. I., Kim H. A. (2005). Correlation between ultrasonographic findings and the response to corticosteroid injection in pes anserinus tendinobursitis syndrome in knee osteoarthritis patients. *Journal of Korean Medical Science*.

[8] Hansen P. A., Willick S. E., Braddom R. L. (2015). Musculoskeletal disorders of the lower limb. *Physical Medicine & Rehabilitation*.

[9] Basford J., Delisa J., Gans B. (1998). Physical agents. *Rehabilitation Medicine: Principles and Practice*.

[10] Fadadu P. P., Mazzola A. J., Hunter C. W., Davis T. T. (2019). Review of concentration yields in commercially available platelet-rich plasma (PRP) systems: a call for PRP standardization. *Regional Anesthesia and Pain Medicine*.

[11] Mazzocca A. D., McCarthy M. B. R., Chowaniec D. M., Cote M. P., Romeo A. A., Bradley J. P. (2012). Platelet-rich plasma differs according to preparation method and human variability. *Journal of Bone and Joint Surgery*.

[12] Yu T.-Y., Pang J.-H. S., Lin L.-P., Cheng J.-W., Liu S.-J., Tsai W.-C. (2021). Platelet-rich plasma releasate promotes early healing in tendon after acute injury. *Orthopaedic Journal of Sports Medicine*.

[13] Liu B., Jeong H. J., Yeo J. H., Oh J. H. (2021). Efficacy of intraoperative platelet-rich plasma augmentation and postoperative platelet-rich plasma booster injection for rotator cuff healing: a randomized controlled clinical trial. *Orthopaedic Journal of Sports Medicine*.

[14] Fan Y., Feng Z., Cao J., Fu W. (2020). Efficacy of extracorporeal shock wave therapy for achilles tendinopathy: a meta-analysis. *Orthopaedic Journal of Sports Medicine*.

[15] Larsson L. G., Baum J. (1985). The syndrome of anserina bursitis: an overlooked diagnosis. *Arthritis & Rheumatism*.

[16] Weiderpass E., Botteri E., Longenecker J. C. (2019). The prevalence of overweight and obesity in an adult Kuwaiti population in 2014. *Frontiers in Endocrinology*.

[17] Gur G., Turgut E., Dilek B., Baltaci G., Bek N., Yakut Y. (2017). Validity and reliability of visual analog scale foot and ankle: the Turkish version. *Journal of Foot and Ankle Surgery*.

[18] Basaran S., Guzel R., Seydaoglu G., Guler-Uysal F. (2010). Validity, reliability, and comparison of the WOMAC osteoarthritis index and Lequesne algofunctional index in Turkish patients with hip or knee osteoarthritis. *Clinical Rheumatology*.

[19] Ritchie D. M., Boyle J. A., McInnes J. M. (1968). Clinical studies with an articular index for the assessment of joint tenderness in patients with rheumatoid arthritis. *Quarterly Journal of Medicine*.

[20] Taheri P., Khosrawi S., Ketabi M. (2017). Investigating the effect of extracorporeal shock wave therapy on reducing chronic pain in patients with pes anserine bursitis: a randomized, clinical- controlled trial. *Advanced Biomedical Research*.

[21] Saba E. K. A. (2022). Efficacy of neural prolotherapy versus local corticosteroid soft tissue injection for treatment of chronic anserine bursitis: a prospective randomized clinical trial. *Ain-Shams Journal of Anaesthesiology*.

[22] Liao C. D., Xie G. M., Tsauo J. Y., Chen H. C., Liou T. H. (2018). Efficacy of extracorporeal shock wave therapy for knee tendinopathies and other soft tissue disorders: a meta-analysis of randomized controlled trials. *BMC Musculoskeletal Disorders*.

[23] Sarifakioglu B., Afsar S. I., Yalbuzdag S. A., Ustaömer K., Bayramoğlu M. (2016). Comparison of the efficacy of physical therapy and corticosteroid injection in the treatment of pes anserine tendinobursitis. *Journal of Physical Therapy Science*.

[24] Yasar M. F., Kurul R., Yaksi E., Aydilek M., Ates Z., Tonuk S. B. (2022). Comparison of the efficacy of corticosteroid and local anesthetic injections combined with physiotherapy in patients with concomitant pes anserine bursitis and knee osteoarthritis: a prospective randomized study. *EJMI*.

[25] Karabaş Ç., Talay Çaliş H., Topaloğlu U. S., Karakükçü Ç. (Jun. 2021). Effects of ultrasound guided leukocyte-rich platelet-rich plasma (LR-PRP) injection in patients with pes anserinus tendinobursitis. *Transfusion and Apheresis Science*.

[26] Rowicki K., Plominski J., Bachta A. (2014). Evaluation of the effectiveness of platelet rich plasma in treatment of chronic pes anserinus pain syndrome. *Ortopedia Traumatologia Rehabilitacja*.

[27] Eyigor C., Eyigor S., Kivilcim Korkmaz O. (2010). Are intra-articular corticosteroid injections better than conventional TENS in treatment of rotator cuff tendinitis in the short run? A randomized study. *European Journal of Physical and Rehabilitation Medicine*.

[28] Bannuru R. R., Schmid C. H., Kent D. M., Vaysbrot E. E., Wong J. B., McAlindon T. E. (2015). Comparative effectiveness of pharmacologic interventions for knee osteoarthritis: a systematic review and network meta-analysis. *Annals of Internal Medicine*.

[29] Ahmed A., Abdelkareem M., Abbas A., Ali W., Gouda W. (2022). Ultrasound, fluoroscopic-guided caudal, lumbar epidural steroid injections and blinding paraspinal lumbosacral steroid injections in patients with low back pain with radiculopathy. *Open Journal of Anesthesiology*.

[30] Gouda W. A., Abbas A. S., Abdel-Aziz T. M. (2023). POS1347. evaluating the efficacy of local corticosteroid injection, platelet-rich plasma, and extracorporeal shockwave therapy in patients with pes anserine bursitis: a pilot randomized, comparative clinical trial. *Annals of the Rheumatic Diseases*.

